# Unraveling the pectinolytic function of *Bacteroides xylanisolvens* using a RNA-seq approach and mutagenesis

**DOI:** 10.1186/s12864-016-2472-1

**Published:** 2016-02-27

**Authors:** Jordane Despres, Evelyne Forano, Pascale Lepercq, Sophie Comtet-Marre, Grégory Jubelin, Carl J. Yeoman, Margret E. Berg Miller, Christopher J. Fields, Nicolas Terrapon, Carine Le Bourvellec, Catherine M.G.C. Renard, Bernard Henrissat, Bryan A. White, Pascale Mosoni

**Affiliations:** Institut National de la Recherche Agronomique (INRA), UR454 Microbiologie, Centre de Clermont-Ferrand/Theix, 63122 Saint-Genès Champanelle, France; Department of Animal and Range Sciences, Montana State University, Bozeman, MT 59718 USA; Department of Animal Sciences, University of Illinois at Urbana-Champaign, Urbana, IL USA; Institute for Genomic Biology, University of Illinois at Urbana-Champaign, Urbana, IL USA; Architecture et Fonction des Macromolécules Biologiques (AFMB), UMR 7257 CNRS, Université Aix-Marseille, 163 Avenue de Luminy, 13288 Marseille, France; INRA, USC 1408 AFMB, 13288 Marseille, France; INRA, UMR408 Sécurité et Qualité des Produits d’Origine Végétale, F-84000 Avignon, France; Université d’Avignon et des Pays de Vaucluse, UMR408 Sécurité et Qualité des Produits d’Origine Végétale, F-84000 Avignon, France; Department of Biological Sciences, King Abdulaziz University, Jeddah, Saudi Arabia

**Keywords:** Pectin degradation, Human gut, *Bacteroides*, Polysaccharide-Utilization Locus, CAZymes, RNA-seq, Mutagenesis

## Abstract

**Background:**

Diet and particularly dietary fibres have an impact on the gut microbiome and play an important role in human health and disease. Pectin is a highly consumed dietary fibre found in fruits and vegetables and is also a widely used additive in the food industry. Yet there is no information on the effect of pectin on the human gut microbiome. Likewise, little is known on gut pectinolytic bacteria and their enzyme systems. This study was undertaken to investigate the mechanisms of pectin degradation by the prominent human gut symbiont *Bacteroides xylanisolvens*.

**Results:**

Transcriptomic analyses of *B. xylanisolvens* XB1A grown on citrus and apple pectins at mid- and late-log phases highlighted six polysaccharide utilization loci (PUL) that were overexpressed on pectin relative to glucose. The PUL numbers used in this report are those given by Terrapon et al. (Bioinformatics 31(5):647-55, 2015) and found in the PUL database: http://www.cazy.org/PULDB/. Based on their CAZyme composition, we propose that PUL 49 and 50, the most overexpressed PULs on both pectins and at both growth phases, are involved in homogalacturonan (HG) and type I rhamnogalacturonan (RGI) degradation, respectively. PUL 13 and PUL 2 could be involved in the degradation of arabinose-containing side chains and of type II rhamnogalacturonan (RGII), respectively. Considering that HG is the most abundant moiety (>70 %) within pectin, the importance of PUL 49 was further investigated by insertion mutagenesis into the *susC-like* gene. The insertion blocked transcription of the *susC-like* and the two downstream genes (*susD-like/FnIII*). The mutant showed strong growth reduction, thus confirming that PUL 49 plays a major role in pectin degradation.

**Conclusion:**

This study shows the existence of six PULs devoted to pectin degradation by *B. xylanisolvens*, one of them being particularly important in this function. Hence, this species deploys a very complex enzymatic machinery that probably reflects the structural complexity of pectin. Our findings also highlight the metabolic plasticity of *B. xylanisolvens* towards dietary fibres that contributes to its competitive fitness within the human gut ecosystem. Wider functional and ecological studies are needed to understand how dietary fibers and especially plant cell wall polysaccharides drive the composition and metabolism of the fibrolytic and non-fibrolytic community within the gut microbial ecosystem.

**Electronic supplementary material:**

The online version of this article (doi:10.1186/s12864-016-2472-1) contains supplementary material, which is available to authorized users.

## Background

The human genome does not encode enzymes for the breakdown of dietary carbohydrates other than sucrose, lactose and a portion of starch [1, 2]. All other complex carbohydrates form dietary fibres that by-pass the absorptive regions of the small intestine and provide a major source of nutrients for the resident gut microbiota. Symbiotic microorganisms that reside in the human large intestine have the capacity to utilize and convert dietary fibres into simple molecules such as the short chain fatty acids acetate, propionate and butyrate that provide an important energy source to the host and additional health benefits [3–7]. Among the main dietary fibres (resistant starches, cellulose, hemicelluloses and pectin), resistant starches have received the greatest scientific focus with nutritional studies aimed at determining the effect of resistant starch consumption on the intestinal microbiota and host health [6, 8, 9]. On the other hand, plant cell wall (PCW) polysaccharide components are much more difficult to study because of their molecular complexity and the difficulty preparing homogenous and pure fractions. This is particularly true for pectin, which is composed of as many as 17 different monosaccharides and more than 20 different linkages [10]. Pectin is made of three major pectic polysaccharides, all containing D-galacturonic acid (GalA). The first one, homogalacturonan (HG), is a linear polymer consisting of 1,4-linked α-D-GalA that may be both methyl-esterified and acetylated. The second polysaccharide, rhamnogalacturonan I (RGI), consists of the repeating disaccharide [/4)- α-D-GalA-(1/2)- α-L-Rha-(1/] to which a variety of different glycan chains (principally α-L-arabinan and arabinogalactan) are attached to the L-rhamnose residues. The third polysaccharide bears the confusing name of rhamnogalacturonan II (RGII), although it is a backbone of HG rather than RG, with complex side chains of rare sugars attached to the GalA residues. Structurally, RGI and HG constitute the ‘backbone’ of pectic polymers. However, another structure has recently been proposed in which HG is a long side chain of RGI [11]. RGI is the most heterogeneous of these three pectic polysaccharides because of its diverse sugar composition and the variation in length of sugar side chains whereas RGII is thought to have a highly conserved structure [11].

It has been reported that around 4 to 5 g of pectins are consumed each day in a normal western diet [11] and that approximately 90 % reach the colon [12]. Yet, there is no clear-cut observation on the impact of pectin on the intestinal microbiota [13] although dietary pectin consumption has been shown to provide health benefits to the host lowering cholesterol and serum glucose level [14, 15], with a possible functional role of intestinal microbiota in these benefits [16]. Likewise, little is known on human intestinal pectin degraders and on the enzyme systems involved in pectin breakdown [13]. Several bacterial isolates belonging to the *Bacteroides* and *Faecalibacterium* genera have been described to be able to grow on pectin as the sole carbon source [17–19]. Because of the molecular complexity of pectin, these bacteria must produce an arsenal of carbohydrate active enzymes (CAZymes; http://www.cazy.org/; [20]). Only one study has investigated pectinolytic enzyme systems in human intestinal bacteria and this was done on *Bacteroides thetaiotaomicron* and *Bacteroides ovatus* using a transcriptomic microarray approach. This study underlined the complexity of this fibrolytic function since several different Polysaccharide Utilization Loci (PUL) were identified in the two species [17]. Furthermore, recent studies have also investigated hemicellulose degradation by *Bacteroides* species and have shown the existence of one and two PUL(s) dedicated to xyloglucan and xylans, respectively [21, 22].

Consequently, the objective of the present study was to further investigate the PCW polysaccharide degradation by the human gut microbiota by focusing on the pectin enzyme system(s) of *Bacteroides xylanisolvens* XB1A, a xylanolytic species belonging to the human core microbiome [23] that exhibits pectinolytic activities.

The genome of *B. xylanisolvens* XB1A encodes 261 glycoside hydrolases (GH), 21 polysaccharide lyases (PL) and 19 carbohydrate esterases (CE) (data from the CAZyme database; http://www.cazy.org/). Examination of the sequence-based family membership of these enzymes suggests that *B. xylanisolvens* XB1A might be able to forage on all major dietary polysaccharides, including starch, fructan, xyloglucan, β-mannan, xylan and pectin in addition to animal and fungal glycans including host’s mucins. Predictions need however to be validated experimentally. For instance, *B. xylanisolvens* harbors CAZymes potentially active on xyloglucan although it does not grow on this polysaccharide [21]. Interestingly *B. xylanisolvens* encodes over 100 genes potentially involved in pectin digestion (Table 1). In addition, *in silico* analyses of the *B. xylanisolvens* XB1A genome recently revealed the existence of approx. 74 Polysaccharide Utilization Loci (PULs) mostly dedicated to dietary fibre degradation [24]. Currently, citrus peel and apple pomace are the major sources of extracted pectin used widely in food industry as functional food ingredient (EU code, E440) [11]. Therefore we chose these two types of pectins to investigate the pectinolytic function of *B. xylanisolvens* using a transcriptomic RNA-seq approach associated with directed mutagenesis.

**Table 1:** List of CAZyme families potentially involved in pectin breakdown and inventory of these CAZymes in *Bacteroides xylanisolvens* XB1A

CAZyme family	Enzyme function	Number of CAZymes	Putative aligosaccharide target within pectins
Glycoside hydrolases (GH)			
GH2	β-galactosidase	23	RGI side chain
GH28	polygalacturonase. rhamno-galaturonase, …	9	HG/RGI/RGII
GH35	β-galactosidase, exo-β-1,4-galactanase …	2	RGI side chain
GH42	β-galactosidase, α-L-arabinofuranosidase	1	RGI side chain
GH43	α-L-arabinofuranosidase, arabinanase …	27	RGI side chain
GH51	α-L-arabinofuranosidase	4	RGI side chain
GH53	endo-β-1,4-galactanase	0	RGI side chain
GH54	α-L-arabinofuranosidase/β-xylosidase	0	RGI side chain
GH78	α-L-rhamnosidase	7	RGI
GH88	d-4,5 unsaturated β-glucuronyl hydrolase	5	RGII side chain
GH93	exo-α-L-1,5-arabinanase	0	RGI side chain
GH95	fucosidase	4	RGII side chain
GH105	unsaturated rhamnogalacturonyl hydrolase	5	HG /RGI
GH106	α-L-rhamnosidase	3	RGI
GH127	α-L-arabinofuranosidase	1	RGI side chain
Polysaccharide lyases (PL)			
PL1	pectate lyase/exo-pectate lyase/pectin lyase	5	HG/RGII
PL3	pectate lyase	0	HG/RGII
PL4	rhamnogalacturonan lyase	0	RGI
PL9	pectate lyase/exopolygalacturonate lyase	1	HG/RGII
PL10	pectate lyase	1	HG/RGII
PL11	rhamnogalacturonan lyase	4	RGI
Carbohydrate esterases (CE)			
CE8	pectin methylesterase	2	HG
CE12	pectin methylesterase/rhamnogalacturonan acetylesterase	4	HG/RGI
CE13	pectin methylesterase	0	HG

## Results

### Upregulation of potential Pectin Utilization Loci in *B. xylanisolvens* XB1A

*B. xylanisolvens* XB1A grows at a rate comparable to that seen with glucose, when commercial citrus and apple pectins were used as sole carbon sources (Table 2). RNA-seq data (Illumina/HiSeq2000) were obtained from the bacterium grown on citrus, apple pectin and glucose at different growth phases (Table 3).

**Table 2:** Growth of *B. xylanisolvens* XB1A and PUL 49 *susC-like* mutant on glucose and pectins

	*B. xylanisolvens* XB1A		PUL 49 *susC* (BXY_31990) Mutant	
Substrate	Rate (∆OD_600_/h)	Density (OD _600_ max)	Rate (∆OD_600_/h)	Density (OD _600_ max)
Glucose	0.25	1.8	0.25	1.8
Citrus Pectin	0.24	1.6	0.06	0.9
Apple Pectin	0.19	1.6	0.18	1.2
Amylase-Treated Apple Pectin	0.16	1.6	0.05	0.8

**Table 3:** Analyses performed from *B. xylanisolvens* XB1A cultures according to substrate and growth phase^a^

	Mid-log Phase		Late-log Phase	
Glucose	RNA-seq	RT-qPCR	RNA-seq	RT-qPCR
Citrus Pectin	RNA-seq	RT-qPCR	RNA-seq	RT-qPCR
Apple Pectin	nd	nd	RNA-seq	RT-qPCR
Amylase-treated Apple Pectin	nd	RT-qPCR	nd	RT-qPCR

^a^Not done

#### Citrus pectin *versus* glucose

**Fig. 1:**
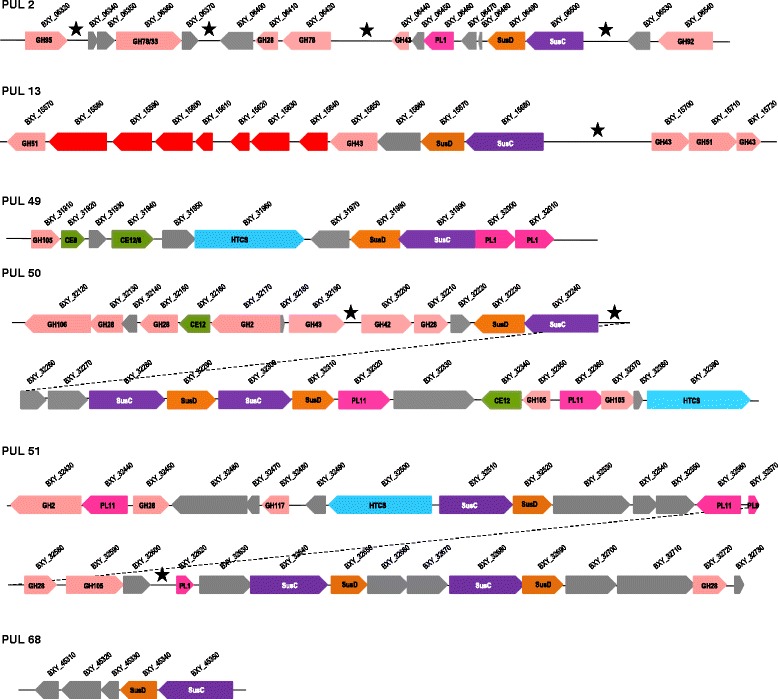
Genomic organization of the six PULs over-expressed on citrus pectin relative to glucose. The color code used for carbohydrate active enzymes highlights the nature of the main functional module: glycoside hydrolase (*light pink*), polysaccharide lyase (*dark pink*) or carbohydrate esterase (*light brown*). PUL marker genes, *susC*- and *susD*-like genes, are represented by purple and orange boxes, respectively, whilst the HTCS regulator gene appears in light blue. Other genes predicted as members of the PULs are shown in grey except genes involved in sugar metabolism in red. Genomic regions containing N stretches and/or unassigned genes are marked with a star

**Fig. 2:**
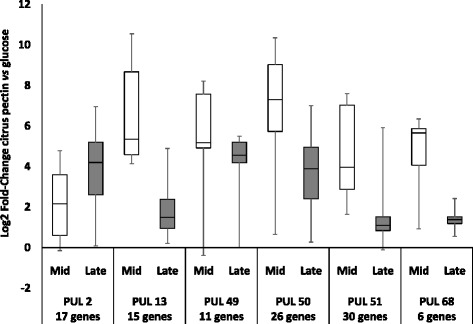
Expression of PULs 2, 13, 49, 50, 51 and 68 on citrus pectin relative to glucose according to growth phase. The distribution of the Log2 Fold-Change of all genes of each PUL is represented in box plots

Expression profiling revealed that 207 and 140 genes were significantly over-expressed (Log2 Fold-change > 3) on citrus pectin at mid- and late-log phase, representing 5.2 and 3.5 % of total genes in the genome, respectively (Additional file 1: Figure S1). Approximately half of these genes belong to the predicted PULs described by Terrapon et al. [24]. The other half encode genes involved mostly in bacterial metabolism (Additional file 1: Figure S2). Inversely, 17 and 14 genes were significantly repressed (Log2 Fold-change < 3) on citrus pectin at mid- and late-log phase, respectively. Most of them belong to PUL 12 (BXY_14320 to BXY_14380) possibly targeting inulin-type fructans and PUL 18 (BXY_19520 to BXY_19610; unknown substrate). Among the 74 PULs predicted from genome analysis [24], 20 PULs were more or less up-regulated on citrus pectin (result based on the average Log2 Fold-change > 2 of each PUL; Additional file 1: Figure S3). Of these induced PULs, we retained the most up-regulated ones (average Log2 Fold-change > 3) which corresponded to six PULs *i.e.* PUL 2 (BXY_06320 to BXY_06540), PUL 13 (BXY_15570 to BXY_15720), PUL 49 (BXY_31910 to BXY_32010), PUL 50 (BXY_32120 to BXY_32390), PUL 51 (BXY_32430 to BXY_32730), and PUL 68 (BXY_45310 to BXY_45350) (Additional file 1: Figure S3 and S4). It occurs that these six PULs contained CAZymes potentially involved in pectin breakdown (Fig. 1, Table 1) and that they were differentially expressed according to the growth phase (Fig. 2). As expected, gene over-expression was higher at mid-log phase than at late-log phase, but interestingly, several genes of PUL 2 (BXY_06320 to BXY_06540) exhibited greater expression at late-log phase than at mid-log phase (Fig. 2; Additional file 1: Figure S4).

#### Apple pectin *versus* glucose

Only 30 genes were overexpressed (Log2Fold-Change ≥ 3) on apple pectin relative to glucose at late-log phase, representing 0.8 % of total genes in the genome (Additional file 1: Figure S1). PUL 71 (BXY_47580 to BXY_47670) was the most up-regulated and this PUL closely resembles the locus encoding the starch utilization system in *B. thetaiotaomicron* (Additional file 1: Figure S2). Among the six PULs identified in this study to be potentially dedicated to citrus pectin degradation, only PUL 49 (BXY_31910 to BXY_32010) and 50 (BXY_32120 to BXY_32390) were up-regulated on apple pectin, but at lower levels than on citrus pectin (Additional file 1: Figure S4). There were very few repressed genes (Additional file 1: Figure S1) and they mainly belonged to PUL 18 (BXY_19520 to BXY_19610) and 65 (BXY_42670 to BXY_42750) whose functions are unknown.

**Fig. 3:**
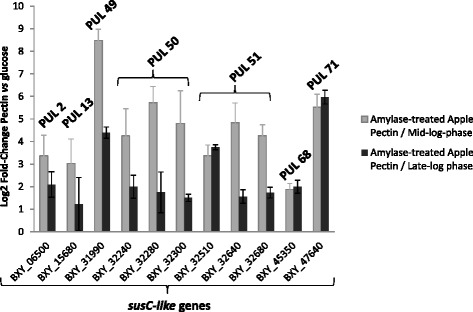
Expression measured by RT-qPCR of *susC-like* genes from PULs 2, 13, 49, 50, 51 and 68 on amylase-treated apple pectin according to growth phase relative to glucose. *B. xylanisolvens* XB1A was grown on amylase-treated apple pectin and harvested at mid- and late-log phase. Each bar represents the mean of three independent experiments

From these RNA-seq data, contamination of apple pectin with starch was suspected and eventually confirmed by analyzing the composition of apple pectin and comparing it to that of the same pectin after amylase treatment and to that of citrus pectin (Additional file 2: Table S1). Apple pectin contained 4.4 % starch while amylase-treated apple pectin and citrus pectin contained 1.3 and 0.7 % starch, respectively. Using amylase-treated apple pectin, the expression of all *susC-like* genes from the six PULs highly induced on citrus pectin (PULs 2, 13, 49, 50, 51 and 68) as well as that of the *susC-like* gene from the putative amylolytic PUL 71 was determined by RT-qPCR at mid- and late-log phase, relative to glucose. The data showed an up-regulation of all *susC-like* genes from the putative pectinolytic PULs except for PUL 68 and only at mid-l og phase (Fig. 3). Over-expression of PUL 71 was still high at mid- and late-log phase.

#### Description of the PULs dedicated to citrus pectin degradation in *B. xylanisolvens* XB1A

PUL 2 (BXY_06320 to BXY_06540) contains 17 genes transcribed from the positive strand (first 5 genes) or the negative strand (last 12 genes) of the genome (Fig. 1). The cluster encodes one *susC/D* homolog pair, six glycoside hydrolases among which five are predicted to be involved in pectin degradation (families GH28, GH33, GH43 and GH78) and one polysaccharide lyase of family PL1 also active on pectin. No regulator was identified but the genomic sequence within this PUL presents several sequencing gaps (series of N) with one particularly large region (3786 nucleotides, BXY_06430) that may contain the missing regulator.

PUL 13 (BXY_15640 to BXY_15720) is highly over-expressed on citrus pectin, especially at mid-log phase. From our RNA-seq data (Additional file 1: Figure S4), this PUL is larger than initially predicted by Terrapon et al. [24]: it should start at BXY_15570 instead of BXY_15640. It contains 16 genes encoding one *susC/D* homolog pair, five glycoside hydrolases potentially active on arabinose-containing oligosaccharides (families GH43, GH51), seven genes involved in sugar transport and metabolism and one gene with unknown function. Five genes of this PUL are also induced on oat-spelt xylan (BXY_15650 to BXY_15700) but at lower levels (2 < Log2Fold-Change < 3) (not shown). As for PUL 2, the regulatory gene(s) could be located in the sequencing gap of 4606 nucleotides between BXY_15680 and BXY_15700.

The three other PULs (49, 50 and 51) are localized in the vicinity of each other on *B. xylanisolvens* XB1A genome [24]. PUL 49 (BXY_31910 to BXY_32010) encodes 11 genes including on the negative strand of the genome the *susC/D-like* genes (BXY_31990/BXY_31980) followed by one gene encoding a protein of unknown function harboring a Fibronectin-III-like domain (FnIII) (Fig. 1). On the positive strand, various CAZyme genes are present: one family GH105 glycoside hydrolase, a family CE8 and a two-domain CE12-CE8 carbohydrate esterase, and two family PL1 polysaccharide lyases. All the CAZyme genes contained in this PUL are described as pectin-specific. PUL 49 also encodes a putative inner membrane-associated sensor-regulator of the hybrid two component system (HTCS) family which is probably involved in PUL 49 regulation. PUL 50 (BXY_ 32120 to BXY_32390) and PUL 51 (BXY_32430 to BXY_32730) correspond to clusters of 26 and 30 genes, respectively and are interrupted by several sequencing gaps (Fig. 1). They both contain three *susC/D* homolog pairs, one putative HTCS sensor-regulator and several CAZyme genes. Thirteen from a total of fourteen CAZymes encoded by PUL 50 and nine from a total of ten CAZymes encoded by PUL 51 are most likely pectinolytic (Table 1).

Lastly, PUL 68 (BXY_43310 to BXY_43350) is also highly over-expressed on citrus pectin, particularly at mid-log phase, but its role in pectin degradation is unclear since it is composed of three genes of unknown function associated to one pair of *susC*/*D* homolog genes (Fig. 2). No regulator was identified in this PUL.

**Fig. 4:**
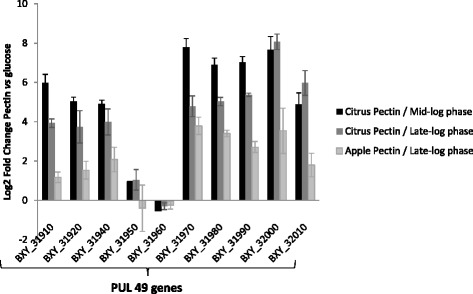
Validation of RNA-seq expression profiles by RT-qPCR targeting PUL 49 genes in citrus or apple pectin conditions relative to glucose. Differences in gene expression between the three conditions *i.e.* citrus pectin/mid-log phase, citrus pectin/late-log phase and apple pectin/late-log phase were significant for all targeted genes (P < 0.01) except for regulation genes (BXY_31950 and BXY_31960), and one PL1_2 gene (BXY_32000)

The RNA-seq expression profiles were validated on one PUL *i.e.* PUL 49 (BXY_31910 to BXY_32010) by RT-qPCR. Gene expression profiles obtained in the different pectin conditions relative to glucose were confirmed except for the two consecutive genes (BXY_32000, BXY_32010) encoding family PL1 polysaccharide lyases (Fig. 4).

### Importance of PUL 49 (BXY_31910 to BXY_32010) in citrus pectin utilization by *B. xylanisolvens* XB1A

In order to confirm the implication of PUL 49 in pectin utilization by *B. xylanisolvens* XB1A, we undertook mutagenesis of the single *susC*-homolog gene (BXY_31990) in this PUL. This gene was chosen because *susC-like* mutants have proven to be useful in establishing the role of PULs in polysaccharide degradation by *Bacteroides thetaiotaomicron* [25]. The preparation of an isogenic deletion mutant using the strategy described by Koropatkin et al. [26] for *B. thetaiotaomicron* was first tried but failed for many reasons, one of them being the very low conjugation efficiency in *B. xylanisolvens* XB1A (see Materials & Methods). Nevertheless a *susC-like* (BXY_31990) insertion mutant was successfully obtained.

**Fig. 5:**
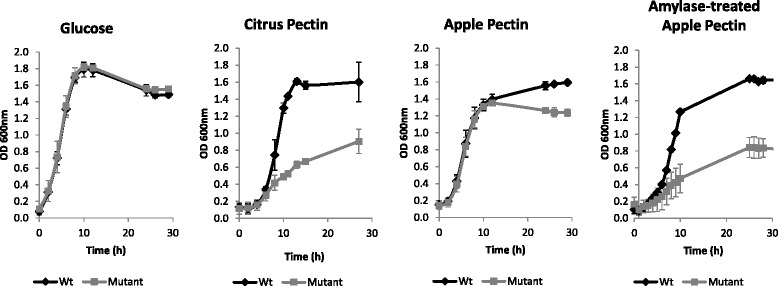
Growth of *B. xylanisolvens* XB1A (Wt) and PUL 49 *susC-like* (BXY_31990) mutant on glucose, citrus pectin, apple pectin and amylase-treated apple pectin. Each curve represents the mean of three independent cultures

**Fig. 6:**
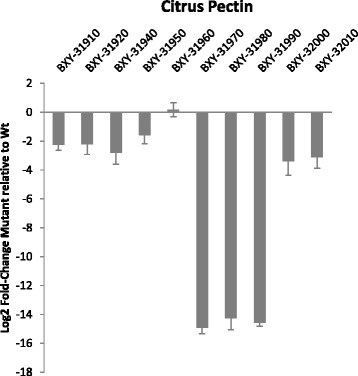
Expression measured by RT-qPCR of PUL 49 genes in PUL 49 *susC-like* (BXY_31990) mutant relative to *B. xylanisolvens* XB1A (Wt). Each strain was grown on citrus pectin and harvested at mid-log phase. Each bar represents the mean of three independent experiments

The PUL 49 *susC-like* mutant exhibited normal colony morphology on agar plates and cells grown in liquid glucose complex medium had a similar shape as wild type cells under the microscope. The growth of the mutant was compared to that of the wild type strain on glucose, citrus pectin, apple pectin and amylase-treated apple pectin (Fig. 5, Table 2). Both strains showed a similar growth on glucose. On citrus pectin, the growth of the mutant was strongly affected in comparison to the wild-type strain. On apple pectin, the difference between mutant and wild-type (Wt) strain was only noticeable at the end of exponential phase. Nevertheless, with amylase-treated apple pectin, the growth of the mutant was affected, as observed on citrus pectin. The expression of PUL 49 genes in the mutant strain was then analyzed upon growth on citrus pectin at mid-log phase (Fig. 6). Except for BXY_31960 (HTCS gene), all PUL 49 genes showed decreased expression in the *susC-like* mutant compared to the Wt strain. Moreover the three consecutive genes transcribed from the negative strand of the genome *i.e. susC-, susD-* and *FnIII-like* genes (BXY_31990, BXY_31980, BXY_31970) were much more repressed (Log2 Fold-Change < -14) than the other PUL genes (-3 < Log2 Fold-Change < -2).

### PUL 49 (BXY_31910 to BXY_32010) synteny with other *Bacteroides* PULs

**Fig. 7:**
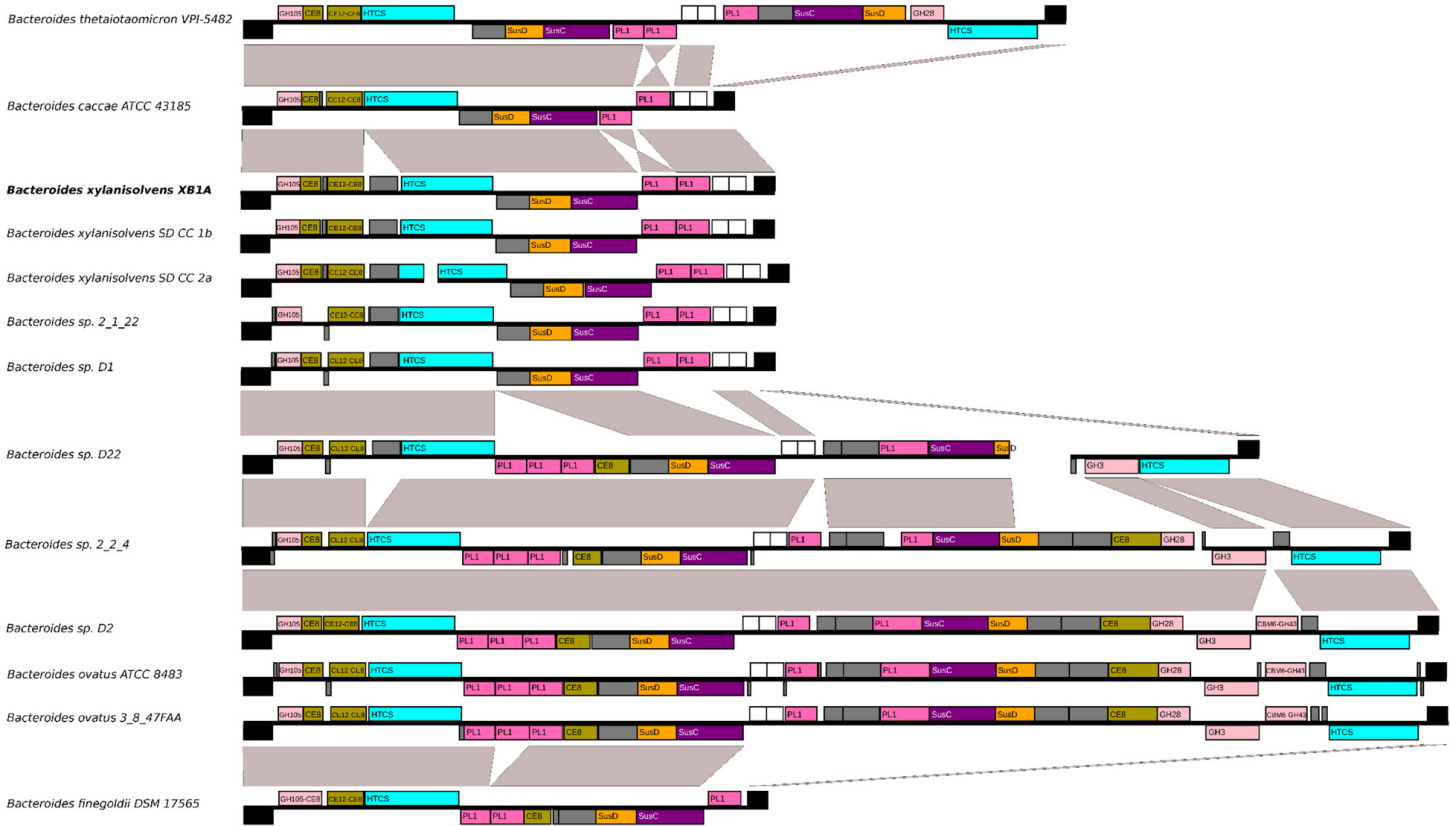
Evolution of the genomic region including XB1A PUL 49 in closely related species. Genomic regions and encoded genes are respectively represented as a central black line with boxes above or below to distinguish strands, with proportionality to genome/gene length. Rearrangements can be visualized thanks to the presence of light-grey polygons between conserved segments of two distinct PUL organizations. The color code used for carbohydrate active enzymes highlights the nature of the main functional module: glycoside hydrolase (*light pink*), polysaccharide lyase (*dark pink*) or carbohydrate esterase (*light brown*). PUL marker genes, *susC*- and *susD*-like genes, are represented by purple and orange boxes, respectively, whilst the HTCS regulator gene appears in light blue. Other genes predicted as members of the PULs are shown in grey. Two adjacent genes that are conserved in most species but only predicted as part of the active loci in the "longest" PUL versions are shown as white boxes. The non-PUL genes immediately flanking the PUL region are shown in black boxes

Given the importance of PUL 49 to citrus pectin utilization by *B. xylanisolvens* XB1A, we searched for analogous PULs in sequenced genomes from other *Bacteroides* species. PULs showing similar organization to PUL 49 were found in *Bacteroides* species phylogenetically close to *B. xylanisolvens i.e. B. ovatus, B. thetaiotaomicron, B. caccae* and *B. finegoldii* (Fig. 7). As a matter of fact, the type strains of these four species are also able to grow on citrus pectin under the same culture conditions as *B. xylanisolvens* XB1A (Additional file 2: Table S2). Several *Bacteroides* strains with no species assignment also harbored an analogous PUL in their genome. Based on the percentage of identity (≥99 %) of the 16S rRNA sequence of these strains with that of species type strains, we suggest that *Bacteroides sp.* 2_1_22, D1, D22 and 2_2_4 belong to *B. xylanisolvens* and *Bacteroides sp.* D2 to *B. ovatus*.

Among the *Bacteroides* spp. harboring homologues of PUL 49, variations were observed, including several rearrangements, such as insertion/deletions of segments comprising one to ten genes. This was true even across strains of the same species. Interestingly, the synteny of the flanking genes is highly conserved across all species despite various PUL organizations. One feature could be retrieved from this PUL comparison among the two phylogenetically close species *B. xylanisolvens* and *B. ovatus*. Indeed, two PUL architectures can be distinguished: the shortest version (approx. 11 genes) found in *B. xylanisolvens* strains XB1A, SD-CC-1b, SD-CC-2a, 2_1_22, D1 displays two to three PL1-encoding genes and a single *susC*/*D*-like and HTCS system, while the longest version (approx.32 genes) found in *B. xylanisolvens* strains as well as in *B. ovatus* strains includes four to five PL1-containing genes, two s*usC*/*D*-like + HTCS systems, and additional CAZyme genes coding for one to two CE8 and one to three GH modules of family 3, 28 and 43 potentially active on pectin.

## Discussion

This study was undertaken to elucidate the pectinolytic function of *B. xylanisolvens* XB1A, knowing that pectin is a natural dietary component found in fruits and vegetables and citrus and apple pectins are widely used in food industry [11].

Citrus pectin has been shown to be rich in GalA (>70 % total sugars) and to be highly methylated, indicating that it consists predominantly of HG [27, 28]. Its major neutral sugars are D-galactose, L-arabinose and L-rhamnose, indicating the presence of RGI branched with neutral sugar side chains like α-L-arabinan and arabinogalactan [28]. The monosaccharide composition as well as the degree of methylation of the commercial citrus pectin used in this study confirmed this description (Additional file 2: Table S1). *B. xylanisolvens* must then deploy enzymes targeting primarily HG and RGI. CAZymes involved in pectin degradation must disrupt esterified groups (mainly methyl groups) and neutral sugar side chains from the backbone, and also cleave the glycosidic bonds within the GalA-containing polysaccharide backbones (HG, RG-I, RG-II) and within the neutral sugar side chains [13].

RNA-seq transcriptomic analysis showed that PULs 2, 13, 49, 50, 51 and 68 play a major role in citrus pectin breakdown (Fig. 1). Taking glucose substrate as reference condition, PUL 49 (BXY_31910 to BXY_32010) and 50 (BXY_32120 to BXY_32390) were the most up-regulated with citrus pectin. PUL 49 composition in CAZyme genes (two PL1, two CEs) suggests that it targets homogalacturonan (Table 1). Homologous PULs were found to be up-regulated on purified HG in *Bacteroides thetaiotaomicron* VPI-5482 (PUL 75) and *Bacteroides ovatus* ATCC 8483 (PUL 100), hence confirming this hypothesis (Fig. 7; http://www.cazy.org/PULDB/) [17]. *B. xylanisolvens* PUL 50 encodes CAZymes targeting L-rhamnose-containing (GH28, GH105, GH106, PL11) and D-galactose-containing oligosaccharides (GH2, GH3, GH42), suggesting that this PUL could be dedicated to RGI. The organization of this PUL presents similarities with a portion of PUL 77 in *B. thetaiotaomicron* VPI-5482 shown to be overexpressed on purified RGI [17]. PUL 51 (BXY_32430 to BXY_32730) was less overexpressed on citrus pectin than PUL 49 and PUL 50. Its composition in CAZyme genes suggests that it encodes enzyme that attack both the HG and RGI pectin backbone (Table 1). PUL 13 (BXY_15570 to BXY_15720) and PUL 68 (BXY_45310 to BXY_45350) were particularly overexpressed at early stages (mid-log phase) of *B. xylanisolvens* XB1A growth while PUL 2 (BXY_06320 to BXY_06540) was upregulated at late stages (late-log phase) of growth. Although it is not possible to propose a role for PUL 68 in pectin breakdown, PUL 13 encodes CAZymes potentially active on neutral sugar side chains since the three GH43 and two GH51 enzymes may target arabinose-containing oligosaccharides. In *B. thetaiotaomicron* VPI-5482, PUL 7 (http://www.cazy.org/PULDB/) whose organization is almost identical to PUL 13 was indeed shown to be induced on arabinans [17]. The fact that PUL 13 (and maybe PUL 68) are overexpressed at early stages of bacterial growth suggests that the targeted oligosaccharides are the most accessible pectin moieties and/or the easiest to hydrolyze. Finally, we assume that PUL 2 targets the most resistant pectin polysaccharide RGII. This assumption is based on the fact that PUL 2 is homologous to PUL 89 which is up-regulated on purified RGII in *B. thetaiotaomicron* VPI-5482 [17]. Actually, PUL 2 is overexpressed at late stages of *B. xylanisolvens* growth, once the easy hydrolysable pectic molecules *i.e.* HG and RGI have been consumed and rendered less available. Also, PUL 2 encodes a GH95 enzyme putatively able to attack the complex side chains of rare sugars in RGII (Table 1), enzyme that is not present in the other PULs.

Like citrus pectin, apple pectin contains more than 70 % of highly-methylated HG [29], and this was confirmed by the analysis of the commercial citrus and apple pectins used in this study. The difference between the two pectins may reside in the rhamnogalacturonans, with longer RGI regions in apple pectin and higher content in neutral sugars [30]. Also, several articles on apple pectin have reported contamination by co-extracted starch [30, 31]. A contamination with 4.4 % starch was found in the apple pectin used in this study and may explain the fact that PUL 71 was the most overexpressed PUL by *B. xylanisolvens* on this substrate. Indeed, this PUL must encode a starch utilization system considering its homology with the starch utilization locus (PUL 66, http://www.cazy.org/PULDB/) described in *B. thetaiotaomicron* [25]. Otherwise, PUL 49 and 50 were also up-regulated with apple pectin, as observed with citrus pectin, although the intensity of the response was lower on apple pectin, probably because the bacterium also consumed starch.

Once treated with amylases, the composition of apple pectin was very similar to that of citrus pectin if we consider their content in GalA, rhamnose, galactose and the degree of methylation. Nevertheless, apple pectin contained two-fold less arabinose, three-fold more glucose and five-fold more xylose than citrus pectin, suggesting a lower extent in arabinan side chains and a residual contamination with starch and PCW polysaccharide moieties. Using this amylase-treated apple pectin, and through the analysis of the relative expression of *susC-like* genes, we validated the induction of five PULs over the six PULs identified with citrus pectin and confirmed their involvement in pectin-utilization by *B. xylanisolvens* XB1A.

Overall, this transcriptomic study using both RNA-seq and RT-qPCR data obtained with two pectin sources and at two stages of bacterial growth, pointed to two overexpressed PULs *i.e.* 49 (BXY_31910 to BXY_32010) and 50 (BXY_32120 to BXY_32390) that we assumed targeted HG and RGI, respectively. Considering that HG is the most abundant moiety within pectin, PUL 49 must therefore play a major role in pectin deconstruction. To confirm this hypothesis, PUL 49 was mutated on the *susC-like* gene, which encodes a membrane TonB-dependent transporter allowing internalization of pectic oligomers within the bacterial periplasm, and hence the mutation is expected to block substrate utilization by the bacterium [25]. The analysis of the mutant in comparison to the wild type strain confirmed our hypothesis since the growth of the mutant on citrus pectin and amylase-treated apple pectin was strongly affected, both in rate and density. However growth was not completely abolished, suggesting that the bacterium could still utilize pectin moieties by means of the other enzyme systems possibly encoded by the other PULs highlighted in our study (PULs 2, 13, 50, 51 and 68). Performing deletion mutagenesis of all putative pectinolytic PULs identified in this study would have been ideal to better comprehend their role but this was not possible because *B. xylanisovens* XB1A was particularly recalcitrant to genetic manipulation. With untreated apple pectin as substrate, the mutant growth was only affected during stationary phase, possibly because it preferentially used contaminating starch and started to hydrolyze pectin only when the medium was depleted in starch. This observation highlights the adaptive capacity of *B. xylanisolvens* in utilizing mixtures of plant polysaccharides. Nevertheless, starch contamination prevented us from comparing thoroughly the RNA-seq transcriptomic profiles of *B. xylanisolvens* on apple and citrus pectins. As a consequence, caution must be taken when using so called “purified” apple pectins when the objective is to screen for pectinolytic bacteria and to analyze pectinolytic function.

To better understand the mutant phenotype, we analyzed PUL 49 (BXY_31910 to BXY_32010) gene expression in the mutant grown on citrus pectin in comparison to the wild type strain. Plasmid insertion into the *susC-like* gene (BXY_31990) blocked the expression not only of that gene but also of the two downstream genes *i.e. susD-like* (BXY_31980) and *FnIII-like* genes (BXY_31970), suggesting that these three genes form an operon. This is supported by short (<20pb) intergenic distances and operon prediction using Rockhopper v. 1.30 [32]. Moreover, except for the gene encoding the sensor/regulator protein (HTCS, BXY_31960), the other PUL 49 genes were also slightly repressed in the mutant. This overall repression is probably due to the fact that the sensor/regulator protein, usually located in the cytoplasmic membrane, was not activated by HG oligosaccharides that could not enter the periplasm through the SusC-like transporter.

Finally, this study showed the architectural complexity of homogalacturonan-targeting PULs in *Bacteroides* which highlights important inter- and intra-species genetic rearrangements. Nevertheless, within the *B. xylanisolvens* species, two distinct PUL architectures were identified with strains harboring a short PUL analogous to PUL 49 (BXY_31910 to BXY_32010), and other strains harboring a long PUL consisting of a PUL 49-like segment and of another PUL segment, as in *B. ovatus*. A question is whether the acquisition or loss of this PUL segment has functional repercussions in terms of efficiency of pectin utilization and/or in terms of adaptability to structurally-different pectins. Only interaction studies between *Bacteroides* species harboring different PULs in response to well characterized (and pure) pectin from various plants will allow us to answer this question. Of course, wider functional and ecological studies are needed to understand how dietary fibers and especially PCW polysaccharides drive the composition and metabolism of the fibrolytic community within the intestinal microbial ecosystem. In the long term, such studies would provide valuable information aiming at maintaining a healthy gut microbiota or rebalancing a disturbed microbiota through diets enriched in specific PCW polysaccharides.

## Conclusions

This study shows the existence of six PULs involved in pectin degradation by *B. xylanisolvens* XB1A, one of them being particularly important in this function. This species deploys a very complex enzymatic machinery that probably reflects the structural complexity of this dietary fibre. Our findings highlight the metabolic plasticity of *B. xylanisolvens* towards non-starch dietary polysaccharides which contributes to its competitive fitness within the human gut ecosystem.

## Methods

### Bacterial strain, media and growth conditions

*B. xylanisolvens* XB1A^T^ (DSM 18836^T^) was grown anaerobically at 37°C in a complex medium containing clarified rumen fluid [33] and 5g/L of citrus peel pectin (Fluka, France), apple pectin (Sigma-Aldrich, France) or glucose. Rumen fluid was collected in the experimental slaughterhouse at INRA, Saint-Genes-Champanelle, France, from animals slaughtered in accordance with the guidelines of the local Ethics Committee and current INRA ethical guidelines for animal welfare (Permit number: 63345001). The media were prepared, dispensed and inoculated by using strictly anaerobic techniques in Balch tubes. A 2.5 % (v/v) inoculum of culture pre-adapted on each substrate was used for inoculation. Bacterial growth was followed by optical density of the culture at 600 nm (OD_600nm_) recorded directly in Balch tubes using a Jenway 6320D spectrophotometer. Three independent cultures were performed for each substrate condition for subsequent transcriptomic analyses (Table 3).

### Pectin analyses

Neutral sugars contained in pectin were analyzed as alditol acetates after acid hydrolysis. Briefly, ten milligram pectin powder were pre-hydrolyzed with 250 μl of 72 % sulfuric acid for 1 h at room temperature [34]. The mixture was then diluted with water to reach a final concentration of sulfuric acid of 1 mol/L. After addition of inositol at 1 g/L (internal standard), pectin was further hydrolyzed in an oven at 100 °C for 3 h. The released sugars were then derivatized to alditol acetates [35] and analyzed as follows: They were injected in a GC-FID HP 5890 Series II instrument (Agilent, Inc, Palo Alto, USA) with a 30 m × 0.25 mm *i.d.* capillary column coated with DB225 MS, film thickness 0.25 μm (J&W Scientific, Agilent, Inc, Palo Alto, USA). The chromatography conditions were: temperature of injection of 250 °C in split mode (ratio 1:25), hydrogen as carrier gas at 45 cm/s, column flow 1.3 ml/min and oven isothermal temperature at 215 °C.

Uronic acids were measured spectrophotometrically by the m-hydroxydiphenyl assay using galacturonic acid as external standard [36].

Methanol was determined by Headspace-GC-MS after pectin saponification using CD_3_OH as internal standard as previously described [37]. The degree of methylation (DM) was calculated as the molar ratio of methanol to uronic acids.

Contaminating starch in pectins was quantified using a colorimetric-enzymatic method (Total Starch HK Assay Kit, Megazyme International Ireland, Bray, Ireland) and expressed in % (w/w) of initial pectin dry matter. The measurements were performed with a SAFAS flx-Xenius XM spectrofluorimeter (SAFAS, Monaco).

To eliminate contaminating starch from apple pectin, pectin was dissolved in water at the concentration of 5 g/L by overnight incubation at 4 °C. The solution was then brought to pH 6 with NaOH 0.1M before starch enzymatic hydrolysis. α-Amylase from *Aspergillus oryzae* (30 U/mg, Sigma-Aldrich, Steinheim, Germany) and amyloglucosidase from *Aspergillus niger* (30-60 U/mg, Sigma-Aldrich, Steinheim, Germany) were added to the apple pectin solution (1U enzyme/g pectin) and incubated at 40 °C for 2 h. After enzyme treatment, pectin was precipitated by addition of ethanol at a final concentration of 80 % (v/v). The solution was then centrifuged (14000g, 10 min, 20 °C) and the pectin pellet was dried by solvent exchange (ethanol 96 % and acetone), followed by an overnight incubation in an oven at 40 °C.

### Preparation of enriched fractions of mRNAs

Total RNAs were isolated from cultures harvested at mid- and late-log phase using a modified guanidinium–phenol–chloroform procedure previously described for rumen fluid [38]. Briefly, bacterial cultures (4 tubes x 8 ml) were centrifuged for 15 min at 3000 g at 4 °C. The pellets were resuspended in 9 ml of a RNA-E solution containing solution D [39], water saturated phenol, sodium acetate 0.2 M pH 4.0 and 2-mercaptoethanol (1:1:0.1:0.007). Cells were then disrupted by bead beating for 1 min with 0.1 g zirconia beads (0.1 mm) followed by a 2 min incubation at 60 °C. These two steps were then repeated once. After addition of 3.75 ml of chloroform, the samples were briefly mixed, incubated for 15 min on ice and centrifuged (12,000 g, 20 min, 10 °C). The RNAs contained in the aqueous supernatants (approximately 6 ml) were precipitated with 0.25 volume isopropanol and washed with 1 volume 75 % cold ethanol in DEPC-treated water. Total RNAs were solubilized in 100 μl of DEPC-treated water. Genomic DNA was removed using the Turbo DNA-Free DNAse (Ambion, France) for 30 min at 37 °C. RNAs were quantified using a ND-2000 NanoDrop spectrophotometer (Nanodrop Technologies, France). Enriched fractions of mRNAs were prepared using the MicrobExpress™ Bacterial mRNA Purification kit (Ambion, France). The high RNA quality and the reduction in 16S and 23S rRNA in enriched fractions of mRNAs were confirmed using an Agilent 2100 Bioanalyser (Agilent technologies, France).

### RNA-seq analyses

cDNA libraries were prepared with 100 ng of mRNA-enriched fractions following the protocols of the Illumina TruSeq Stranded RNA-seq Sample Preparation Kit. The final libraries had an average fragment size of ∼ 250 bp and were quantified by qPCR before being sequenced with an Illumina HiSeq 2000 instrument on a single lane in paired end reads. A total of approx. 8 to 9 million paired reads per sample was obtained (Additional file 2: Table S3). Quality filtering and adapter trimming were performed with Trimmomatic v0.30 [40] using Illumina TruSeq3 adapter sequences for adapter clipping.

The *B. xylanisolvens* XB1A genome (GenBank accession NC_021017.1) was indexed using novoindex, and reads aligned with novoalign v. 3.00.05 (http://www.novocraft.com) against the indexed genome. For downstream gene expression analysis only the aligned R1 reads were used; these were extracted from the paired-end alignment file using samtools v0.1.19 [41] using the bitwise flag for the first read for a read pair.

Gene counts were determined for the aligned data using featureCounts v. 1.4.3-p1 [42] and the NCBI GFF3 feature file (obtained from the NCBI FTP site Dec. 2013).

Differential gene expression was performed using R 3.0.0 using edgeR/limma [43, 44]. Samples were assessed for potential outliers based on counts using normalized counts (RPKM) that would affect downstream analyses. From this analysis we determined that samples mRNA7 and 10 were problematic and thus removed them from the analysis (Additional file 2: Table S3). Counts per million (CPM) mapped reads were calculated per gene; genes with more 1 CPM in three or more samples were retained in the final edgeR analysis. Samples were normalized using edgeR’s TMM normalization. A simple generalized linear model was generated using the aforementioned filtered data from all remaining samples, and simple contrasts based on carbon source and growth stage were used to determine genes differentially expressed under the conditions shown. A separate coordinate analysis was performed using Rockhopper v. 1.30 [32] on all again using data from NCBI obtained as previously mentioned. Results from this analysis were primarily used to find potential groups of genes that may be expressed as a single transcriptional units or operons.

### Reverse transcription (RT) followed by quantitative PCR (qPCR)

Total RNAs (1 μg) were reverse-transcribed into cDNAs using random hexamer primers (Invitrogen, France) and 200 U SuperscriptII Rnase H^−^ reverse transcriptase (Invitrogen, France) according to the procedure supplied with the enzyme. For each RNA sample, a negative RT (no addition of reverse transcriptase) was performed and used as a negative control in subsequent qPCRs.

The relative expression of 10 target genes of PUL 49 in the citrus pectin (mid and late-log phases) and apple pectin (late-log phase) culture conditions versus glucose condition at the corresponding growth phases was performed by quantitative PCR using a Rotor-Gene Q (Qiagen, France) and QuantiFast SYBR Green PCR Mastermix (Qiagen, France) using supplier’s instructions. The designed specific primers are listed in Additional file 2: Table S2. The fold change in gene expression (pectin *versus* glucose) was calculated from 3 biological replicates (+ two technical replicates) according to Livak and Schmittgen [45] using the 16S rRNA reference gene for normalization. Log2 fold-change at mid- and late-log phase were considered as significantly different at *p* < 0.01 (Student's t-test).

### Mutagenesis

An internal fragment (890pb) of the PUL 49 *susC-like* gene was cloned into the pGERM suicide vector containing *E. coli* (*bla*) and *Bacteroides sp.* (*ermG*) selective markers [46] (Additional file 2: Table S4). The resulting construct was transformed into the conjugative *E. coli* WM3064 strain which is auxotrophic for diaminopimelic acid (DAP). The *E. coli* donor strain WM3064 was grown aerobically at 37 °C in Luria broth (LB) supplemented with DAP (100 μg/ml) and ampicillin (50 μg/ml) until it reached an OD_600_ of 0.2. The *B. xylanisolvens* XB1A recipient was grown anaerobically at 37 °C in glucose complex medium (see above) until it reached an OD_600_ of 1. A 2 ml mixture of equal volumes of donor and recipient cultures (1:1 ratio) was centrifuged, supernatant discarded and 100 μl of the mating mix was placed onto a HAWP filter (0.22 μm pore size) on top of glucose complex medium agar plate supplemented with DAP. After aerobic overnight incubation, the plate was transferred into an anaerobic station (Jacomex, France) and the bacteria on the filter were suspended in 2 ml of glucose complex medium. After 5 h anaerobic incubation at 37 °C, 100 μl cell suspension was spread on glucose complex medium agar plates supplemented (mutant selection) or not (control) with erythromycin (25 μg/ml). After 4 days anaerobic incubation, erythromycin resistant colonies were picked and used for genomic DNA extraction. Plasmid insertion into the target gene was then verified by PCR using primers targeting junction regions between pGERM and PUL 49 *susC-like* gene (Additional file 2: Table S4). A low conjugation efficiency (approx. 10^−8^) was observed with *B. xylanisolvens* XB1A even after defining optimized conjugation conditions. As a matter of comparison, conjugation was a hundred times more efficient with *B. thetaiotaomicron* (approx. 10^−6^).

The expression levels of ten PUL 49 genes in the mutant relative to the wild type strain after growth on citrus pectin at mid-log phase (biological triplicates) were measured by RT-qPCR as described above.

## Availability of supporting data

The data sets supporting the results of this article are included within the article and its additional supplementary file. The transcriptome data are available in GEO datasets at NCBI (http://www.ncbi.nlm.nih.gov/) under the accession number GSE74379.

## Additional files

Additional file 1: Figure S1. Percentage of induced or repressed genes in *B. xylanisolvens* XB1A genome in response to citrus or apple pectin (relative to glucose). **Figure S2.** Induced genes in *B. xylanisolvens* XB1A genome in response to citrus or apple pectin (relative to glucose) which are possibly involved in sugar or energy metabolism or which have unknown function. **Figure S3.** *B. xylanisolvens* XB1A PUL expression in response to citrus or apple pectin relative to glucose. **Figure S4.** Induced genes in *B. xylanisolvens* XB1A genome in response to citrus or apple pectin (relative to glucose) that are potentially involved in pectin degradation. (XLSX 49 kb) Additional file 2: Table S1. Composition of the commercial citrus and apple pectins used in this study. **Table S2.** Growth of *Bacteroides* species on citrus pectin under the same culture conditions as for *B. xylanisolvens* XB1A (DSM18836^T^). **Table S3.** Primers used for relative RT-qPCR targeting PUL 49 genes. **Table S4.** RNA-seq mapping assessment. **Table S5.** Primers used for directed mutagenesis into PUL 49 *susC-like* gene (BXY_31990). (DOCX 24 kb)

## Abbreviations

CAZyme: carbohydrate active enzyme
HG: homogalacturonan
PCW: plant cell wall
PUL: polysaccharide utilization locus
RGI: type I rhamnogalacturonan
RGII: type II rhamnogalacturonan
