# Erratum to: Unraveling the pectinolytic function of *Bacteroides xylanisolvens* using a RNA-seq approach and mutagenesis

**DOI:** 10.1186/s12864-016-2758-3

**Published:** 2016-06-06

**Authors:** Jordane Despres, Evelyne Forano, Pascale Lepercq, Sophie Comtet-Marre, Grégory Jubelin, Carl J. Yeoman, Margret E. Berg Miller, Christopher J. Fields, Nicolas Terrapon, Carine Le Bourvellec, Catherine M.G.C. Renard, Bernard Henrissat, Bryan A. White, Pascale Mosoni

**Affiliations:** Institut National de la Recherche Agronomique (INRA), UR454 Microbiologie, Centre de Clermont-Ferrand/Theix, Saint-Genès Champanelle, 63122 France; Department of Animal and Range Sciences, Montana State University, Bozeman, MT 59718 USA; Department of Animal Sciences, University of Illinois at Urbana-Champaign, Urbana, IL USA; Institute for Genomic Biology, University of Illinois at Urbana-Champaign, Urbana, IL USA; Architecture et Fonction des Macromolécules Biologiques (AFMB), UMR 7257 CNRS, Université Aix-Marseille, 163 Avenue de Luminy, Marseille, 13288 France; INRA, USC 1408 AFMB, Marseille, 13288 France; INRA, UMR408 Sécurité et Qualité des Produits d’Origine Végétale, Avignon, F-84000 France; Université d’Avignon et des Pays de Vaucluse, UMR408 Sécurité et Qualité des Produits d’Origine Végétale, Avignon, F-84000 France; Department of Biological Sciences, King Abdulaziz University, Jeddah, Saudi Arabia

## Erratum

After publication of the original article [1], it came to the authors’ attention that a funding source received by B. Henrissat had been accidently omitted from the Acknowledgements. The following sentence should have been included in the original article:

This work was supported in part by a grant to BH: European Union’s Seventh Framework Program (FP/2007/2013)/European Research Council (ERC) Grant Agreement 322820.
