# Supplementary material for: Unraveling the pectinolytic function of Bacteroides xylanisolvens using a RNA-seq approach and mutagenesis
Source: BMC Genomics. 2016 Feb 27;17:147. doi: 10.1186/s12864-016-2472-1 (PMC4769552; doi:10.1186/s12864-016-2472-1)
Supplement: Additional file 2: Table S1. — Composition of the commercial citrus and apple pectins used in this study. Table S2. Growth of Bacteroides species on citrus pectin under the same culture conditions as for B. xylanisolvens XB1A (DSM18836T). Table S3. Primers used for relative RT-qPCR targeting PUL 49 genes. Table S4. RNA-seq mapping assessment. Table S5. Primers used for directed mutagenesis into PUL 49 susC-like gene (BXY_31990). (DOCX 24 kb) [file 12864_2016_2472_MOESM2_ESM.docx]

**Table S1: Composition of the commercial citrus and apple pectins used in this study**

| **Samples** | **Rha^d^** | **Fuc^e^** | **Ara^f^** | **Xyl^g^** | **Man^h^** | **Gal^i^** | **Glc^j^** | **UA^k^** | **MeOH^l^ (DM %)^m^** | **Starch^n^ (%)** |
| --- | --- | --- | --- | --- | --- | --- | --- | --- | --- | --- |
| Citrus pectin^a^ | 8.2 ± 0.5 | 0 | 28.9 ± 1.2 | 2.4 ± 0.4 | 0 | 54.8 ± 1.3 | 8.4 ± 1.3 | 544.1 ± 14.0 | 78 ± 1 (79 ± 2) | 0.7 ± 0.2 |
| Apple pectin^b^ | 7.7 ± 0.2 | 0 | 12.0 ± 0.1 | 7.5 ± 0.7 | 8.9 ± 0.5 | 43.0 ± 0.2 | 134.9 ± 7.6 | 461.6 ± 55.8 | 62 ± 1 (75 ± 9) | 4.4 ± 0.5 |
| Apple pectin^c^ (amylase-treated) | 8.0 ± 0.1 | 0 | 13.0 ± 0.7 | 10.8 ± 0.6 | 0 | 60.5 ± 3.4 | 25.8 ± 2.2 | 581.9 ± 54.8 | 78 ± 5 (74 ± 12) | 1.3 ± 0.3 |

^a^Citrus pectins (Fluka, France), ^b^Apple pectins (Sigma, France), ^c^Apple pectins (same batch) treated with amylases (see Methods), ^d^Rhamnose, ^e^Fucose, ^f^Arabinose, ^g^Xylose, ^h^Mannose, ^i^Galactose, ^j^Glucose, ^k^Uronic acids, ^l^Methanol, ^m^Degree of methylation, ^n^Starch.

Composition is given in mg/g initial dry matter, except for DM and starch that are given in percentage of initial dry matter. Each value is the mean of three determinations ± standard deviation, except for starch (n=6).

| **Table S2**: Growth of *Bacteroides* species on citrus pectin under the same culture conditions as for *B. xylanisolvens* XB1A (DSM18836^T^) | | |
| --- | --- | --- |
|  |  |  |
|  | Rate (DOD600/h) | Density (DO600 max) |
| *Bacteroides thetaiotaomicron* DSM2079^T^ | 0.13 | 1.5 |
| *Bacteroides caccae* DSM 19024^T^ | 0.13 | 1.3 |
| *Bacteroides finegoldii* DSM17565^T^ | 0.06 | 1.1 |
| *Bacteroides ovatus* DSM1896^T^ / ATCC8483 | 0.05 | 1.0 |

**Table S3**: Primers used for relative RT-qPCR targeting PUL 49 genes

| **Gene ID** | **Primers** | **Sequences (5’-3’)** | **Product size** |
| --- | --- | --- | --- |
| **BXY_31910** | GH105 fwd  GH105 rev | CCGGAGTGGTGATAAGGGTA  CCAATCCGTTCTTGTCCACT | 173 |
| **BXY_31920** | CE8 fwd  CE8 rev | GTCCGCGCTTTTATGGATTA  CCGTATAGGTGCGGAAGGTA | 184 |
| **BXY_31940** | CE12 /CE8 fwd  CE12 /CE8 rev | GGAATGGTACTTCCCGGATT  GGCCGAACTGGATAAAGACA | 154 |
| **BXY_31950** | ATPase fwd  ATPase rev | AACTGTCGTTTCCTCGGCTT  AGTAGAGGGAGCGGTGACAT | 171 |
| **BXY_31960** | HTCS fwd  HTCS rev | GTCCTGCTTTCCATCCGTGA  TGCCGAAGCAGCTTCACTAA | 153 |
| **BXY_31970** | FnIII fwd  FnIII rev | GAATCTGCCCAACCGTCTAA  GCGTGTCTCCTCTTCGGTAG | 174 |
| **BXY_31980** | susD fwd  susD rev | CCGTTCTAGCGAGTTGTTCC  TGGAACCGTCTACCTTGGTC | 161 |
| **BXY_31990** | susC fwd  susC rev | GAATGCCCCAAGCGTTATTA  TTTTGTGGTCGTAGCTGCTG | 150 |
| **BXY_32000** | PL1_(1)_ fwd  PL1_(1)_ rev | ATGGTGCGGCTGATTACGAT  CACGCTCAAAAGCCATACGG | 186 |
| **BXY_32010** | PL1_(2)_ fwd  PL1_(2)_ rev | AGCAGATGATGGGATGCCTG  GTTTCCCGGTGCAGTTTGTC | 160 |

**Table S4**: RNA-seq mapping assessment^a^

| RNAseq Sample ID | Substrate Growth phase | Total reads^b^ (R1) | Reads after trimming & rRNA filtration^c^ | Mapped reads to XB1A genome (% Mapped)^d^ | |
| --- | --- | --- | --- | --- | --- |
| RNA4 | Citrus pectin Late Log phase | 8 951 672 | 5 306 548 | 5 223 948 | (98.2) |
| RNA5 |  | 8 687 919 | 3 176 095 | 2 983 819 | (97.1) |
| RNA6 |  | 7 996 868 | 2 126 075 | 2 034 653 | (96.1) |
| RNA7 | Apple pectin Late Log phase | 8 204 076 | 1 364 600 | 1 323 382 | (96.8) |
| RNA8 |  | 8 868 771 | 3 313 658 | 3 200 368 | (96.4) |
| RNA9 |  | 8 081 670 | 5 392 501 | 5 269 005 | (97.5) |
| RNA10 | Glucose Late Log phase | 8 654 647 | 2 606 780 | 1 869 664 | (71.2) |
| RNA11 |  | 8 291 051 | 3 378 074 | 2 849 220 | (84.3) |
| RNA12 |  | 8 776 019 | 3 356 441 | 2 656 015 | (79.1) |
| RNA19 | Citrus pectin Mid Log phase | 9 402 417 | 5 950 755 | 5 544 519 | (93.2) |
| RNA20 |  | 8 217 085 | 4 601 070 | 4 260 694 | (92.6) |
| RNA21 |  | 9 101 064 | 4 789 397 | 4 326 343 | (90.3) |
| RNA22 | Glucose Mid Log phase | 8 513 539 | 5 801 500 | 5 392 191 | (92.9) |
| RNA23 |  | 9 211 035 | 6 395 006 | 5 992 997 | (93.7) |
| RNA24 |  | 9 300 496 | 5 670 484 | 5 203 812 | (91.8) |

^a^The genome (GenBank accession [NC_021017.1](http://www.ncbi.nlm.nih.gov/nuccore/479162165)) of *Bacteroides xylanisolvens* strain XB1A^T^ (DSM 18836^T^) is complete but contains sequence gaps (series on N) generating incomplete ORFs that were not automatically annotated. In particular, N stretches were found in the region encoding 16S and 23S rRNA. Because of these unassigned gene regions, mapping of trimmed reads onto XB1A genome generated relatively low mapping percentages. As a consequence, rRNA reads were removed before mapping.

^b^Average read length was 100 nt

^c^Based on quality control, trimming was done by head-cropping 15nt from initial reads. rRNA filtering was performed by mapping (with Bowtie 2) trimmed reads onto a rRNA FASTA file of *Bacteroides xylanisolvens* SD-CC-1b retrieved from SILVA database (http://www.arb-silva.de/). The unmapped reads (corresponding to mRNA essentially) were then used for mapping.

^d^Despite trimming and rRNA-filtering, mapping percentages remained lower than 95% for some samples, probably because of genome sequencing gaps as explained above. For RNA 7 and 10, genome coverage was insufficient and these samples were discarded from subsequent analysis.

**Table S5:** Primers used for directed mutagenesis into PUL 49 *susC* gene (BXY_31990)
